# Evaluation of arthroscopic treatment of posterior shoulder instability

**DOI:** 10.1590/1413-78522015230300540

**Published:** 2015

**Authors:** José Carlos Garcia, Lucas Russo Maia, Juliano Rocha Fonseca, José Luís Amim Zabeu, Jesely Pereira Myrrha Garcia

**Affiliations:** 1 Advanced Study Center on Orthopedics and Neurosurgery, Hospital Santa Catarina, São Paulo, SP, Brazil; 2 Pontifícia Universidade de Campinas, Campinas, SP, Brazil

**Keywords:** Joint instability, Shoulder dislocation, Arthroscopy, Shoulder joint/injuries

## Abstract

**OBJECTIVE:**

To provide data for the analysis of arthroscopy as a method of surgical treatment for shoulder and discuss its actual indications and preliminary results.

**METHODS:**

We evaluated 15 patients submitted to reverse Bankart arthroscopic surgery. We used the UCLA (University of California at Los Angeles) score to measure the results before surgery and 12 months thereafter.

**RESULTS:**

The average UCLA score changed from 26.67±0.25 (SD 0.97) before surgery to 34.20±0.53 (SD 2.04) after surgery. The effectiveness of surgery was 93%. In five cases loose bodies were found. A patient undergoing remplissage was evaluated separately. The data did not change after 24 months post-surgery.

**CONCLUSION:**

The arthroscopic treatment of posterior shoulder instability and posterior dislocation of the shoulder has been proved feasible and results in our series followed the same trends as in the literature. *Level of Evidence III, Transversal Retrospective Study.*

## INTRODUCTION

There no agreement in the literature regarding the first description of the posterior shoulder instability. Some authors believe that White^1 ^was the first to describe it in 1741, others believe it was Astley Cooper in 1839.^2^^,^ 3 Since the first descriptions up to the 90s of the 20^th^ century, Malgaine´s series^4^ in 1855 was the one which had the highest number of cases, 37 patients. This is not a common condition, reported in only 2-5% of shoulder dislocations,^3^ with often difficult diagnosis and controversial option treatments.^5^

Biomechanical studies conducted to clarify this pathology have pointed the posterior capsule and the inferior posterior glenohumeral ligament as important later stabilizers of the shoulder.^5^ Posterior labral injury, reverse Bankart and ligament avulsion have been associated with instability of this region, especially in contact athletes.^6^^-^ 9

It is usual to confuse posterior instability of the shoulder with multidirectional instability. The detailed history, clinical examination and appropriate imaging studies are required in order to minimize diagnostic errors.^3^

For the treatment of posterior instability various techniques have been described: bone block such as the reverse Eden-Hybbinette, glenoid osteotomy; surgeries similar to McLaughlin´s; ligament reconstruction, such as reverse Bankart and reverse Putti-Platt; combined techniques and arthroplasty.^10^^,^ 11

Even with the large amount of existing techniques, the surgical treatment of posterior instability has reached a failure rate of about 30% to 50%.^5^ However, recent studies have shown high success rates (around 90%)^12^^,^ 13 with arthroscopic methods.

We aim with this study to contribute to the analysis of arthroscopy as one of the techniques of choice for treatment of posterior shoulder instability and to detail anatomical aspects that indicate or contraindicate the procedure.

## MATERIALS AND METHODS

The procedures were approved by the Research Ethics Committee of PUC-Campinas on 04/06/2011 (Protocol number: 0096/11). All volunteers signed the Free and Informed Consent (FIC), in accordance with Resolution 466/12.

A retrospective analysis of 21 patients treated for dislocation and posterior instability of the shoulder between January 2003 and May 2010 was performed. Of these, four had fixed dislocation impaction of the humeral head on the posterior glenoid. Of these four patients, two have undergone bone graft, but follow up was lost; one patient with bone loss of the humeral head greater than 40% aged over 60 years underwent McLaughlin arthroplasty surgery, and the last patient was subjected to arthroscopic surgery. The remaining 17 patients had recurrent posterior instability and underwent arthroscopic treatment with advancement of the labrum or reverse Bankart surgery; of those 17, we lost follow-up of two patients, who were excluded from the study.

**Figure 1.**
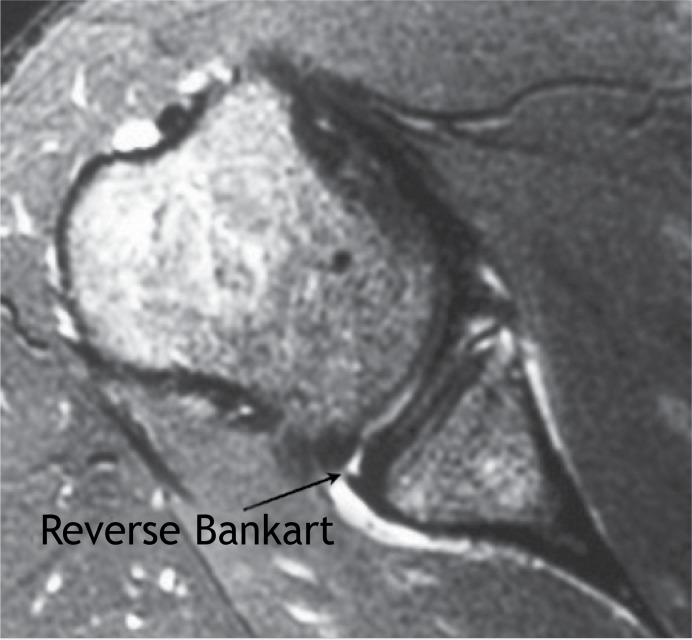
Magnetic Resonance Image of posterior labral injury.

We analyzed patients treated with arthroscopic Bankart reverse technique which met the following inclusion criteria: patients evaluated preoperatively, with at least 12 months postoperatively and followed up for at least 24 months after surgery, aged 18 years or older, with mature skeleton, without mental disabilities or incapacities, with symptoms of posterior instability proven by physical examination, Jerk Test, and additional MRI scans. (Figure 1)

### Surgical Technique

Patients were placed in the beach chair position under general anesthesia associated with brachial plexus block. The arthroscope was inserted at the rear portal. An anterior portal is made and the arthroscope is transferred to this portal. With this view you can detail the injuries of the posterior part of the shoulder.

**Figure 2.**
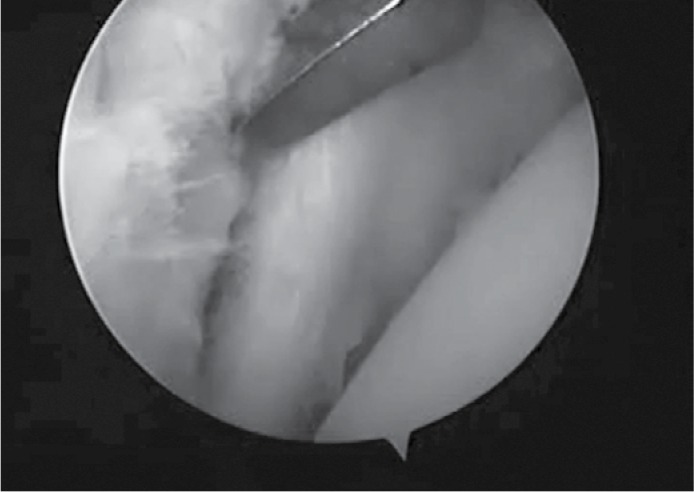
Posterior labral injury.

**Figure 3.**
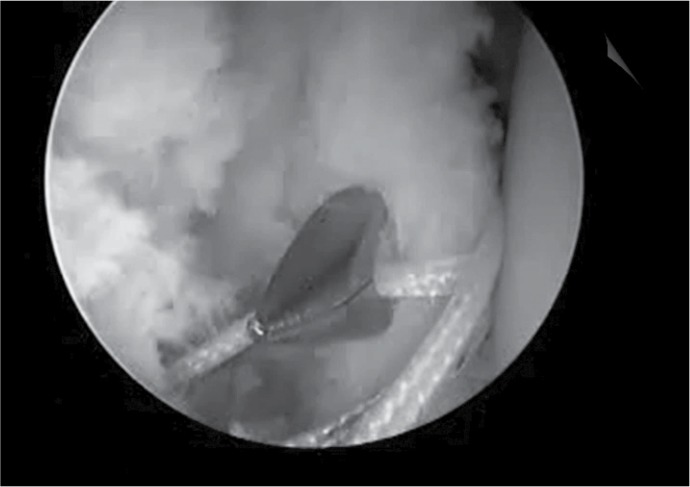
Posterior suture with anchor.

Patients with posterior capsular redundancy also showed medial capsular rise to the labrum and / or labral hypogenesis, in these patients the posterior surface of the scapula next to the glenoid was bloodied and the capsule + labrum residue were both primed and sutured to the posterior glenoid with the aid of anchors, as in the Bankart technique for anterior instability. In patients with posterior labral injury (Figure 2) reverse Bankart arthroscopic surgery and suture anchors were also carried out. (Figure 3) For the anchor insertion a posterolateral portal was made, just below the posterolateral angle of the scapula. All handling and suturing were made through the posterior portal. Anchors should not be inserted through the posterior portal, because the angle of the glenoid version in most cases is not favorable.

Postoperatively patients used for three weeks with a simple sling cushion or abduction triangle to maintain the position of external rotation of the shoulder. After this period the immobilizer has been used for two weeks at night, then physical therapy started.

The 15 patients undergoing arthroscopic treatment with labrum advance and plication or reverse Bankart surgery were evaluated before surgery, at baseline and 12 months after surgery, using functional scale and UCLA (University of California at Los Angeles) quality of life score obtaining, thus, the averages. Losses and related injuries have also been reported.

Statistical evaluation was made by tests respecting the characteristics of the curves, assuming statistical significance of 0.05. The sample size was calculated by interim analysis. 

## RESULTS

From January 2003 to May 2010 17 reverse Bankarts arthroscopic were performed in patients with posterior instability of the shoulder. Two patients had incomplete data on their baseline and loss of follow-up.

Regarding the other 15 patients, 11 had traumatic injuries and four atraumatic origin, 11 on the right side and four on left side, 12 men and three women with a mean age of 28 years old (range 18-36).

The evaluation of patients was performed to treat with the quality of life test and specific function for shoulder surgery of UCLA. The average UCLA score before surgery was 26.66 (range 25-28) and 34.2 (range 27-35) postoperatively. The mean follow-up was 30.26 months at the time of the assessment, since there were no recurrences, except for the patient below. (Table 1)

**Table 1.** Data pre and post operative.

Patients	Age (years old)	Side	Traumatic (T) Atraumatic (A	UCLA pre	UCLA post	Follow up months
1	30	R	T	25	35	49
2	35	L	T	27	34	46
3	18	R	A	26	35	39
4	29	R	A	26	34	39
5	41	R	T	26	35	35
6	33	R	T	26	35	32
7	19	R	A	28	35	30
8	23	R	T	26	35	29
9	19	R	T	27	27	25
10	36	L	T	26	35	26
11	28	R	T	28	35	25
12	25	L	T	28	35	25
13	34	R	T	26	34	23
14	18	L	A	28	35	18
15	26	R	T	27	34	13
	27.6			26.7	34.2	30.266667

One patient did not improve after surgery, keeping the symptoms post-operatively.

**Figure 4.**
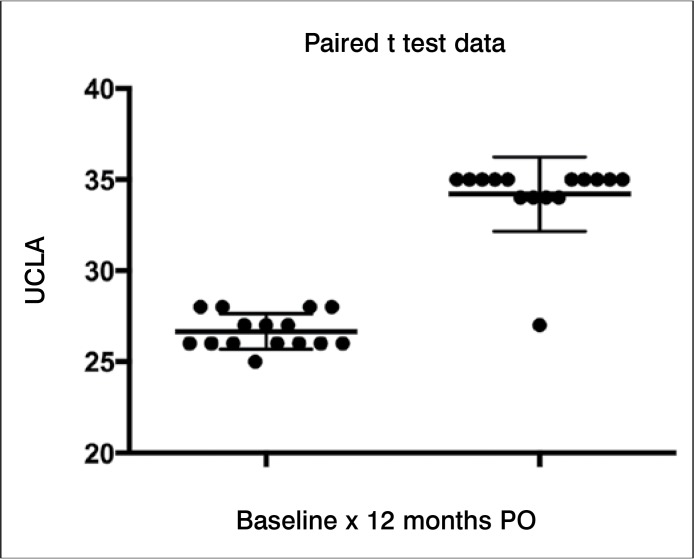
Pre and postoperative assessment

Regarding the statistical evaluations, average baseline was 26.67 ± 0.25 (SD 0.97), post-surgical evaluations showed average of 34.20 ± 0.53 (SD 2.04). (Figure 4) The comparison was made by the Wilcoxon paired test because the data were negative for normality tests. The *p *value was less than 0.0001, showing probability of type 1 error less than 1/10,000.

The sample size in the interim analysis, admitting a difference of 4 points with p <0.001 and statistical power of 99% and using the standard deviation calculated on the baseline, n equals to 14.87, moreover, according to Peto-Haybittle´s rule which assumes that when *p* value is less than 0.001 there is no need for more individuals in the sample to reach a final conclusion of the assessment.^14^ Therefore, the sample size was adjusted to reach a reliable outcome.

The technique showed effectiveness of 93.33%, one case had failed and the pre and postoperative UCLA score did not change.

Regarding safety, in five cases intra-articular loose bodies were identified. There was no neurovascular injury. The data did not change over 24 months after surgery.

## DISCUSSION

Due to anatomical features, such as lack of time between the infraspinatus and supraspinatus muscles and the anteversion of the glenoid, there are fewer degrees of freedom of provocative movements of posterior dislocation from previously; therefore that instability is less common.^9^ Its symptoms can range from severe decrease of external rotation to simple pain to provocative movements.^3^

It is important to differentiate later inveterate dislocation (or posterior instability with bone loss) from posterior instability of the shoulder.^9^^,^ 15 This instability can produce symptoms like pain from dislocation or subluxation to provocative maneuvers, which generally reduce spontaneously and relatively painlessly. The range of motion is maintained. Posterior instability can cause posterior subluxation in the Jerk test, though it is more common to observe only pain during the test. With the patient anesthetized one can usually perceives subluxation posterior to maneuvers, therefore it can be a differential diagnosis of shoulder pain, especially in patients with a history of previous trauma or instability feelings. These symptoms often emphasize in elevation adduction and shoulder internal rotation. In some patients with athletic function and dysplastic posterior labrum later instability can occur, and in such cases, the initial approach should be made by strengthening external shoulder rotators and improve proprioception. This has been noted that particularly in throwing athletes, with satisfactory results by conservative methods.^9^

The posterior shoulder inveterate dislocation can be very painful and be confused with frozen shoulder, the range of motion is highly decreased, especially the external rotation of the shoulder and supination of the forearm.^3^ Non differentiation of these diseases has caused confusion and misinterpretation of data in the literature.^5^^,^ 9 ^,^ 15

Treatment of instability should correct the reverse Bankart injury^5^^,^ 9 or promote advancement and plication of the posterior capsule of the glenoid rim, creating a labrum like with neoanchors.^15^ The inveterate posterior dislocation should be treated with procedures to enable the bone defect caused by injury not to make a lever with the glenoid, promoting a new dislocation. For the treatment of this injury there are several options open; more recently we successfully used in one case McLaughlin^16^ surgery modified by Krackhardt et al.^17^ This surgery is an arthroscopic method of remplissage^18^ that works similarly to McLaughlin's procedure.

We have, therefore, sought to individualize posterior instability in relation to the inveterate posterior dislocation of the shoulder because they have different characteristics and treatments.

The instability can be associated with bone loss, and with recurrence and increased bone injury it could be treated as a dislocation. We do not agree with the statistics that lead to more than 90% of solving to the arthroscopic treatment and only 50% to 70% for open surgery.^5^^,^ 13 On the contrary to some authors´ opinions,^5^ we believe that the interpretation of these works may be wrong, because the injuries treated in the literature are different and incomparable.

The success rates of arthroscopic surgery for posterior shoulder instability are similar to Bankart arthroscopic surgery for anterior instability. However, there is no surgery currently described for posterior bone loss with the same effectiveness of Bristow-Latarjet surgery to anterior bone instabilities.

Our series showed similar effectiveness to previous studies in the treatment of posterior shoulder instability. These results suggest that the mechanisms of posterior instability are similar to anterior instability, where the posterior capsule and the subsequent ligaments act as a network, giving support^10^ and proprioceptive sensitivity to the posterior transfer of the humeral head.

We have used the UCLA scale because it assesses pain, discomfort, limitations, range of motion, function, strength and satisfaction after surgery, being a scale that manages to combine quality of life and function. Other scales such as Rowe, which assess instability consider recurrence, limitation and functional loss, were declined as our functional losses for rotation and elevation were insignificant and we did not observe any level of residual instability. The only patient who shoed loss at surgery had its score unchanged and we found more appropriate to include him in the percentage of treatment effectiveness, thus, keeping the method of choice to treat. Another cause to override Rowe, Wosi or Oxford scales is that the vast majority of patients with posterior instability had pain and not a sense of instability, so the baseline of this scale would not reflect the actual situation of patients. 

As for the position, although all surgeries have been performed in the beach chair position, we believe that when the posterior structures are addressed, the lateral position presents better visibility.

As limitations of this work, studies are comparative with the baseline, therefore, their results will reflect only if surgery is effective or not, but it does not mean that it is more effective than other surgical procedures. This study is retrospective, unblinded and with no possibility of randomization. However, presently there is no other arthroscopic surgical technique that enables a double-blind randomized study for posterior instability of the shoulder.

## CONCLUSION

Arthroscopic treatment of posterior instability of the shoulder and posterior shoulder dislocation is feasible and the results of our series followed the same trends of the literature. This suggests the reliability, effectiveness and safety of arthroscopy in the treatment of dislocation and posterior shoulder instability; however different types of injuries (capsuloligamentous/osseous) may be involved in the pathology and for each type of injury there should be a specific approach.

## References

[1] Loebenberg MI, Cuomo F (2000). The treatment of chronic anterior and posterior dislocations of the glenohumeral joint and associated articular surface defects. Orthop Clin North Am.

[2] Cooper A (1839). On the dislocations of the os humeri upon the dorsum scapulae and upon fractures near the shoulder joint. Guys Hosp Rep.

[3] Steinmann SP (2003). Posterior shoulder instability. Arthroscopy.

[4] Malgaigne JF (1855). Traité des fractures et des luxations.

[5] Savoie FH, Field LD (1997). Arthroscopic management of posterior shoulder instability. Oper Tech Sports Med.

[6] Blasier RB, Soslowsky LJ, Malicky DM, Palmer ML (1997). Posterior glenohumeral subluxation: active and passive stabilization in a biomechanical model. J Bone Joint Surg Am.

[7] Abrams JS (2003). Arthroscopic repair of posterior instability and reverse humeralglenohumeral ligament avulsion lesions. Orthop Clin North Am.

[8] Safran O, Defranco MJ, Hatem S, Iannotti JP (2004). Posterior humeral avulsion of the glenohumeral ligament as a cause of posterior shoulder instability. A case report. J Bone Joint Surg Am.

[9] Bottoni CR, Franks BR, Moore JH, DeBerardino TM, Taylor DC, Arciero RA (2005). Operative stabilization of posterior shoulder instability. Am J Sports Med.

[10] O'Brien SJ, Neves MC, Arnoczky SP, Rozbruck SR, Dicarlo EF, Warren RF (1990). The anatomy and histology of the inferior glenohumeralligament complex of the shoulder. Am J Sports Med.

[11] Sperling JW, Pring M, Antuna SA, Cofield RH (2004). Shoulder arthroplasty for locked posterior dislocation of the shoulder. J Shoulder Elbow Surg.

[12] Provencher MT, Bell SJ, Menzel KA, Mologne TS (2005). Arthroscopic treatment of posterior shoulder instability: results in 33 patients. Am J Sports Med.

[13] McIntyre LF, Caspari RB, Savoie FH 3rd (1997). The arthroscopic treatment of posterior shoulder instability: two-year results of a multiple suture technique. Arthroscopy.

[14] Pocock SJ (1992). When to stop a clinical trial. BMJ.

[15] Wolf EM, Eakin CL (1998). Arthroscopic capsular plication for posterior shoulder instability. Arthroscopy.

[16] McLaughlin HL (1952). Posterior dislocation of the shoulder. J Bone Joint Surg Am.

[17] Krackhardt T, Schewe B, Albrecht D, Weise K (2006). Arthroscopic fixation of the subscapularis tendon in the reverse Hill-Sachs lesion for traumatic unidirectional posterior dislocation of the shoulder. Arthroscopy.

[18] Purchase RJ, Wolf EM, Hobgood ER, Pollock ME, Smalley CC (2008). Hill-sachs "remplissage": an arthroscopic solution for the engaging hill-sachs lesion. Arthroscopy.

